# Supplementary material for: Transcriptomic, Hormonomic and Metabolomic Analyses Highlighted the Common Modules Related to Photosynthesis, Sugar Metabolism and Cell Division in Parthenocarpic Tomato Fruits during Early Fruit Set
Source: Cells. 2022 Apr 22;11(9):1420. doi: 10.3390/cells11091420 (PMC9102895; doi:10.3390/cells11091420)
Supplement: Supplementary file 1 [file cells-11-01420-s001.zip › Supplementary Figures S1¿CS9.pdf]

Supplementary Figures

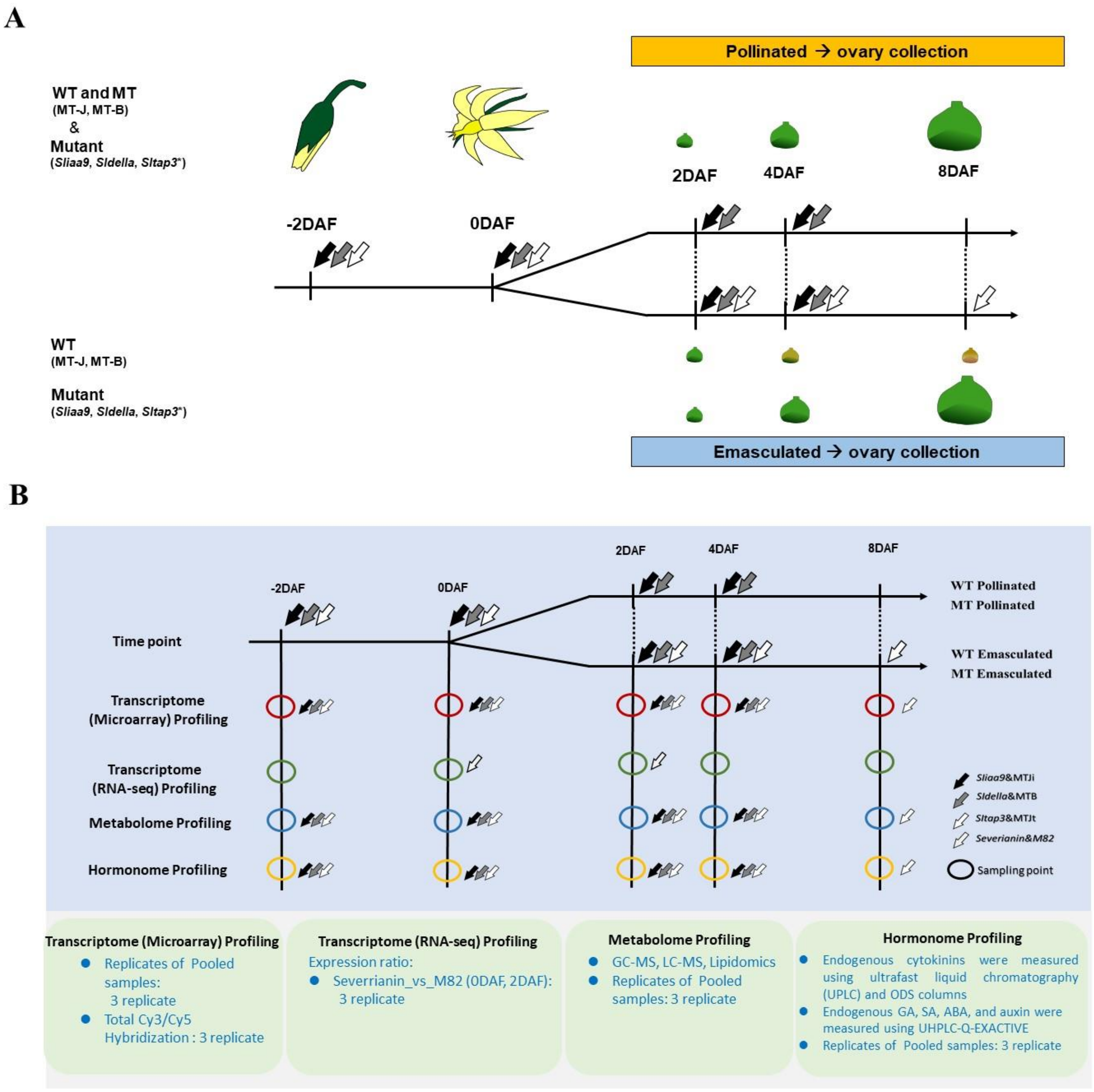

Figure S1. Experimental design for growing conditions (A) and analysis of transcriptomic, metabolomic and hormonome alternations. (B) Aassociation with fruit set in the three mutants, Severianin and the corresponding wild types.

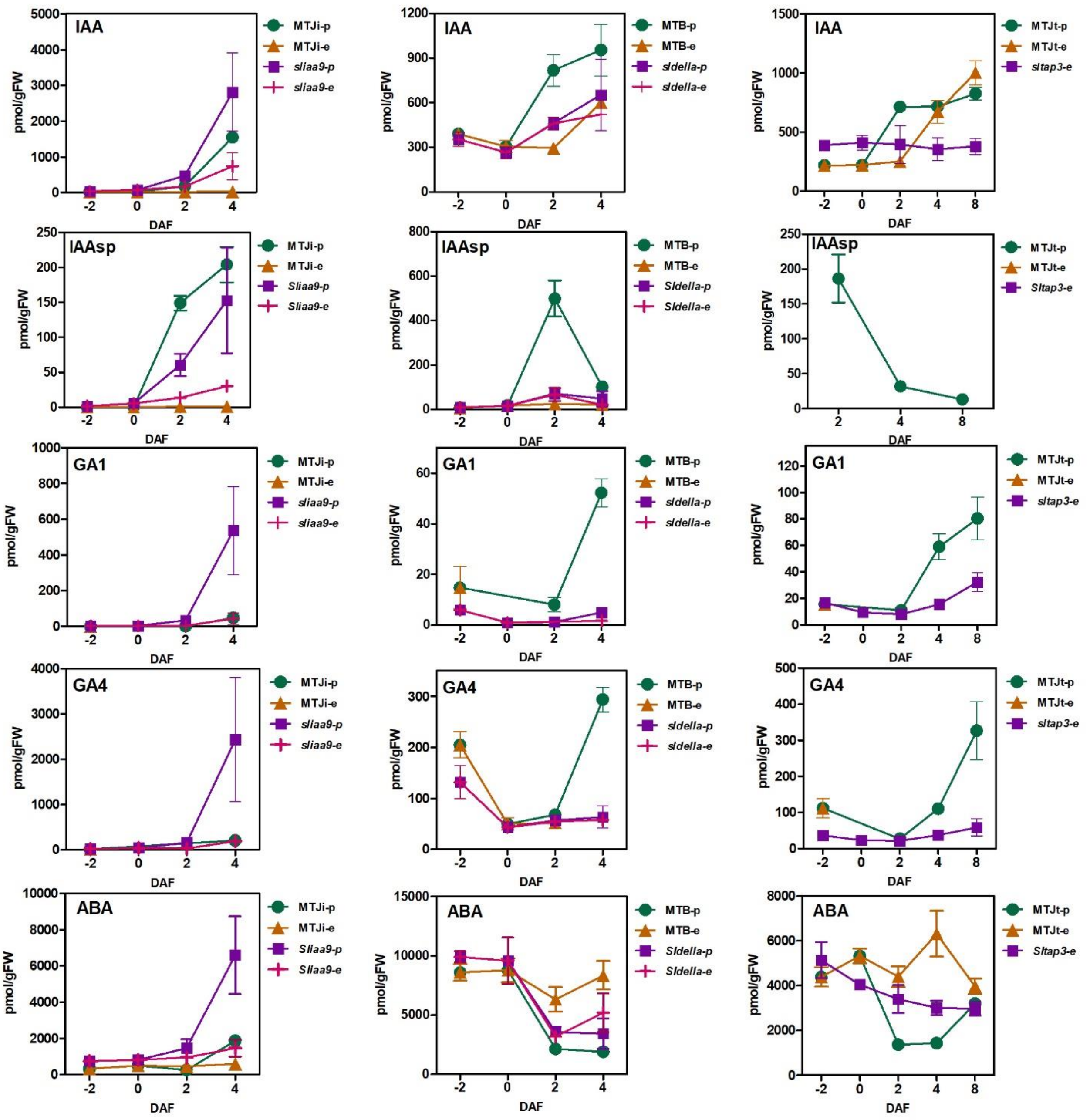

Figure S2. Accumulation patterns of the detected hormones and hormone derivatives during fruit setting in MTJ/*sliaa9*, MTB/*sldella* and MTJ/*sltap3*.

Abbreviations: IAA, indole-3-acetic acid; IAAsp, indoleacetylaspartate; GA, gibberellin; ABA, abscisic acid.

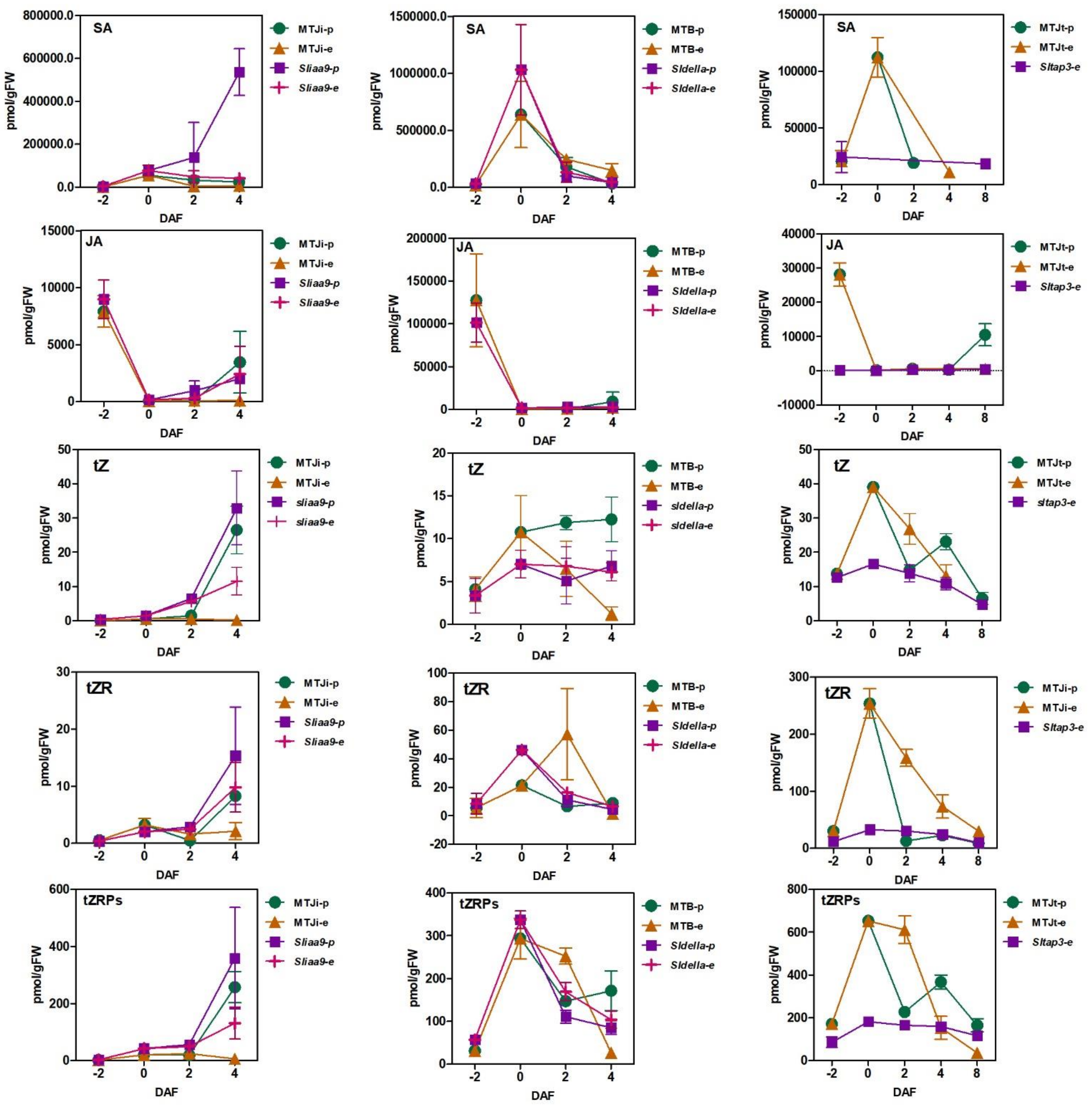

Figure S2 (continued).

Abbreviations: SA, salicyric acid; JA, jasmonic acid; tZ, *trans*-zeatin; tZR, tZ riboside; tZRPs, tZR-5′-monophosphate.

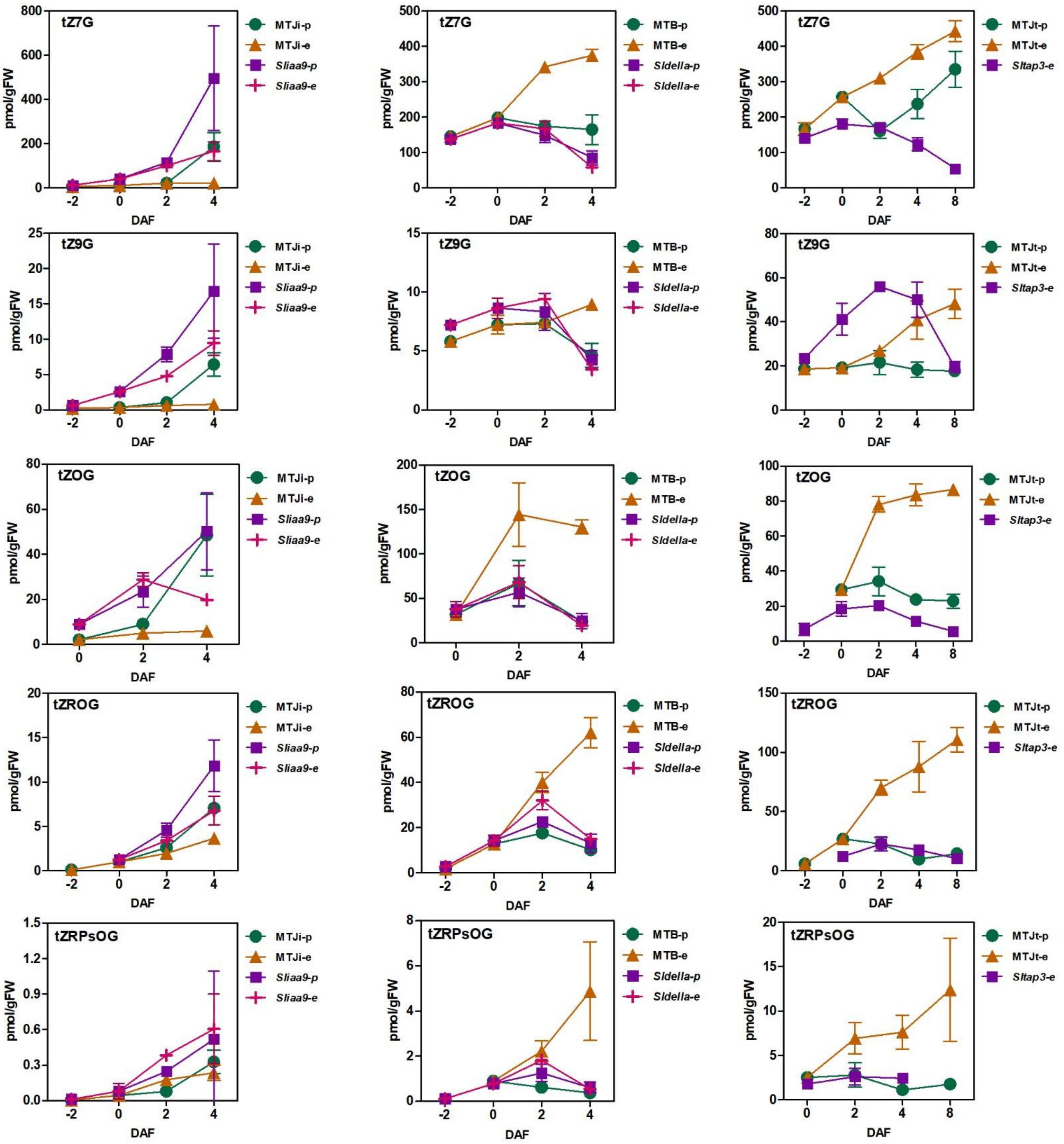

Figure S2 (continued).

Abbreviations: tZ7G, *trans*-zeatin-7-glucoside; tZ9G, *tZ*-9-glucoside; tZOG, tZ-*O*-glucoside; tZROG, tZ-riboside-*O*-glucoside; tZRPsOG, tZR 5′-phosphates-*O*-glucoside.

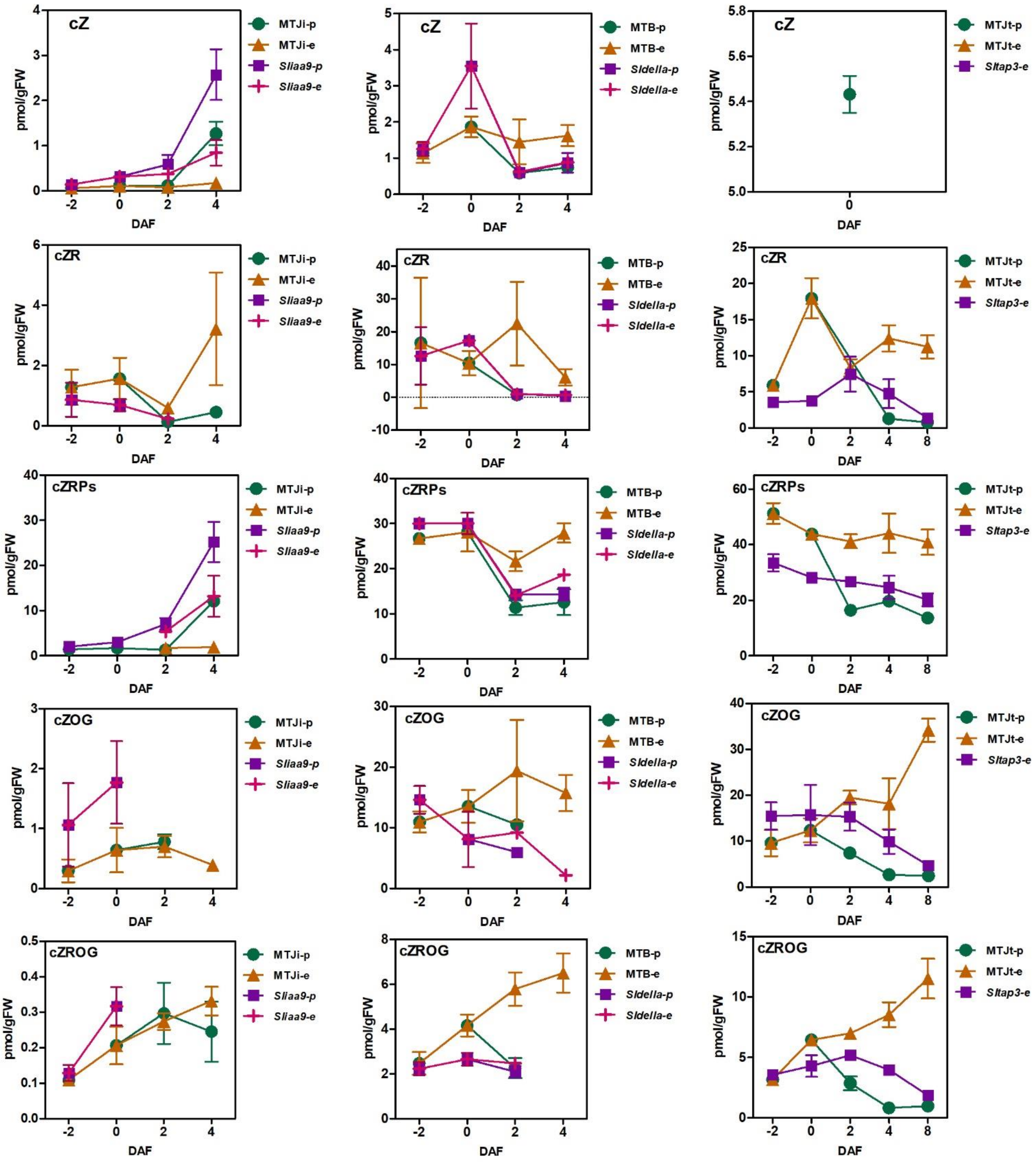

Figure S2 (continued).

Abbreviations: cZ, *cis*-zeatin; cZR, cZ riboside; cZRPs, cZR 5′-phosphates; cZOG, cZ-*O*-glucoside; cZROG, cZR-*O*-glucoside.

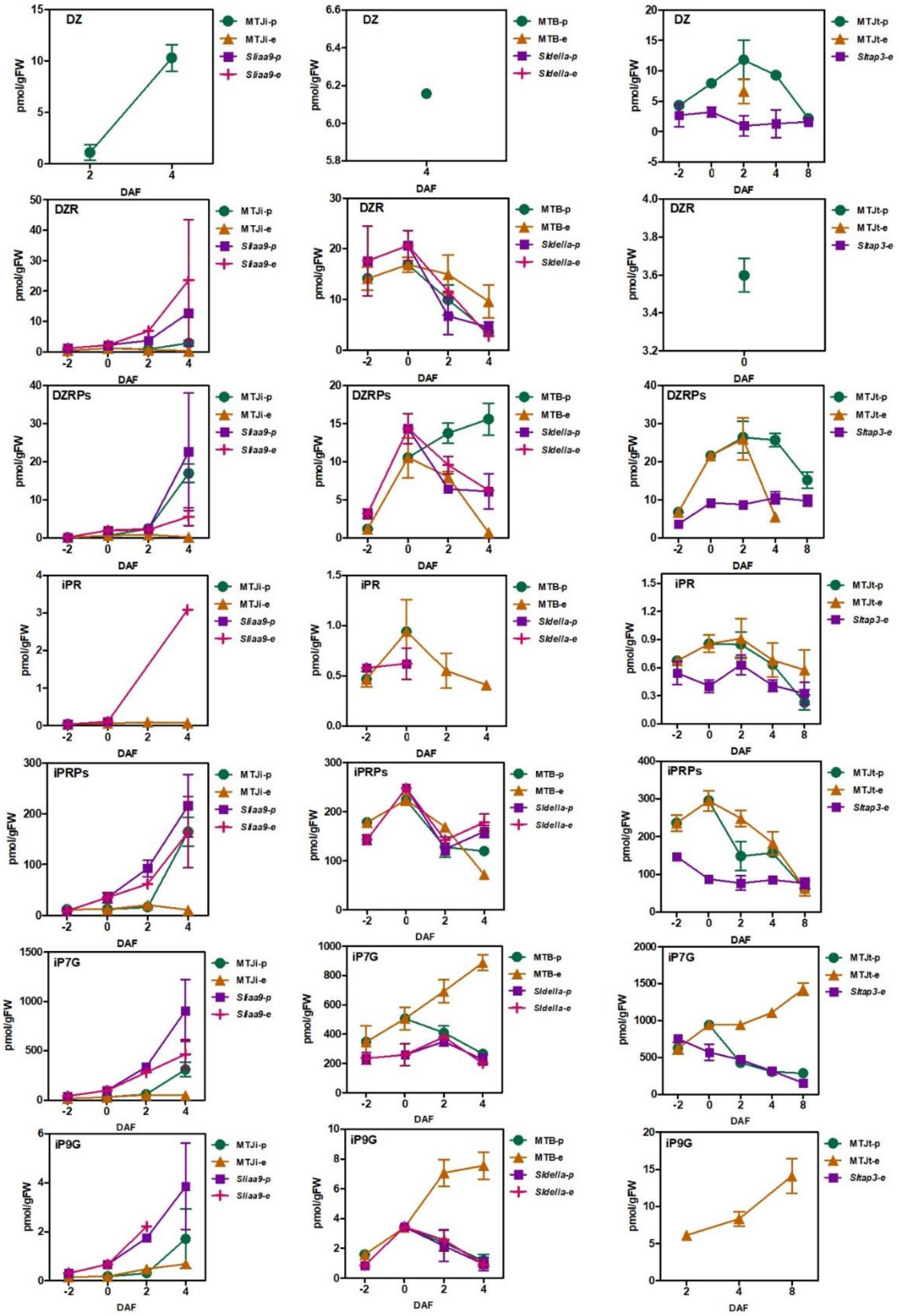

Figure S2 (continued).

Abbreviations: DZ, dihydrozeatin; DZR, DZ riboside; DZRPs, DZR 5′-monophosphate; iPR, isopentenyladenosine; iPRPs, iPR 5′-phosphates; iP7G, N6-isopentenyladenine-7-glucoside; iP9G, N6-isopentenyladenine-9-glucoside.

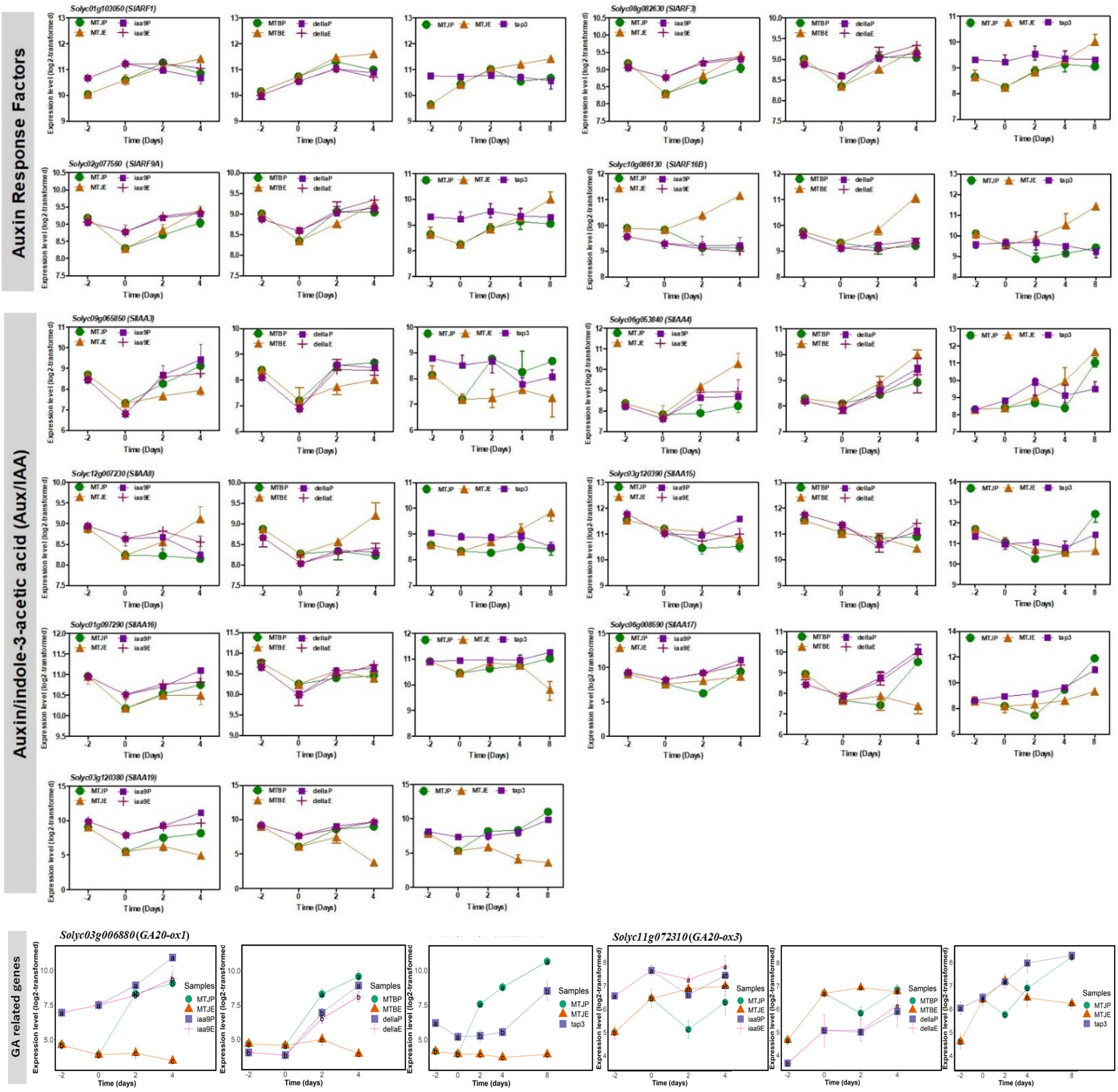

Figure S3. Alternations of auxin and GA related genes during fruit setting in MTJ/*sliaa9*, MTB/*sldella* and MTJ/*sltap3*.

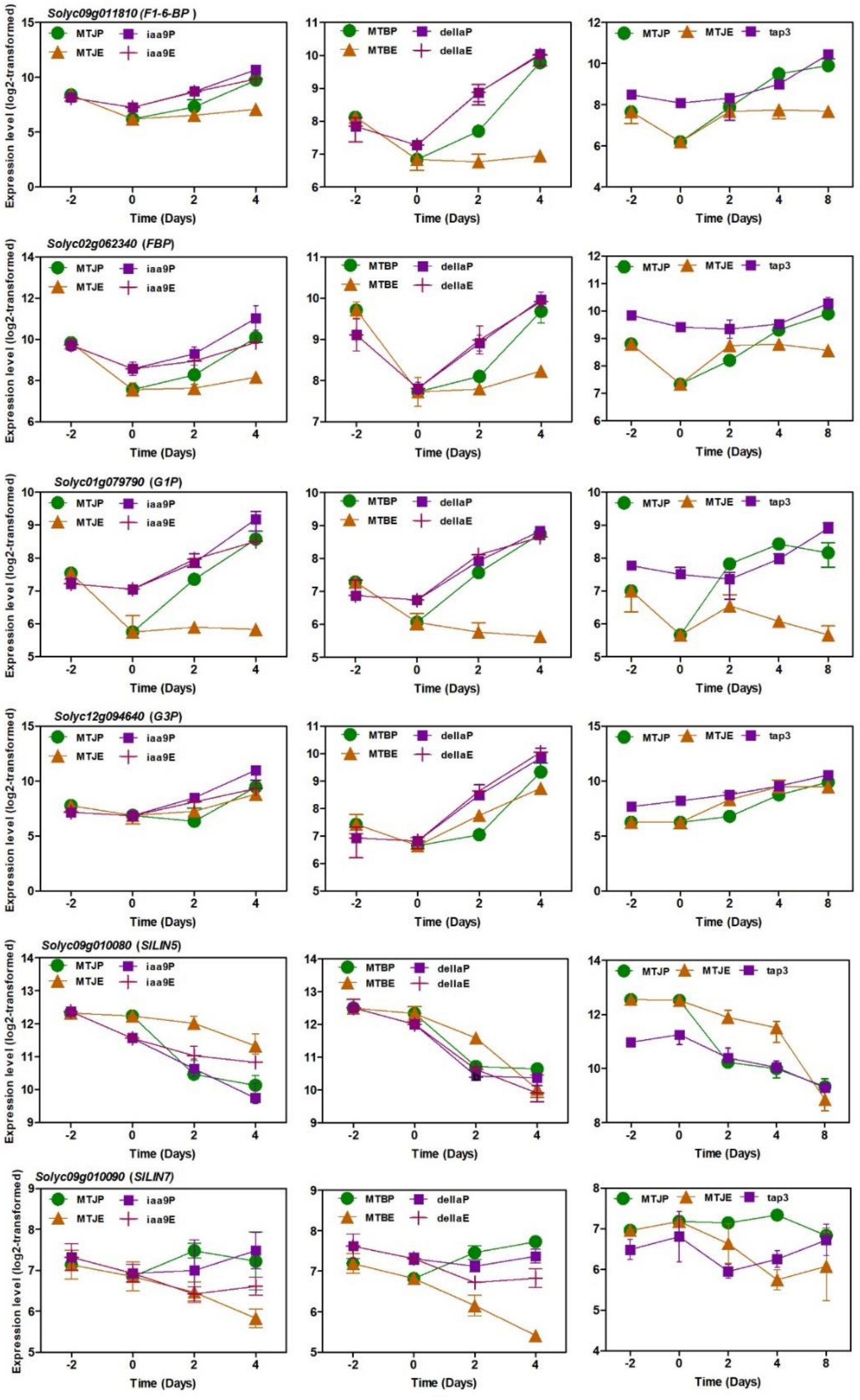

Figure S4. Changes of the genes related to carbohydrate metabolic proces during fruit set in MT and WT with and without pollination**.**

Abbreviations: F1-6-BP, Fructose-1,6-bisphosphatase; FBP, Fructose-bisphosphate aldolase; G1P, Glucose-1-phosphate adenylyltransferase; G3P, Glyceraldehyde-3-phosphate dehydrogenase; SlLIN5, SlLIN7, cell-wall invertase.

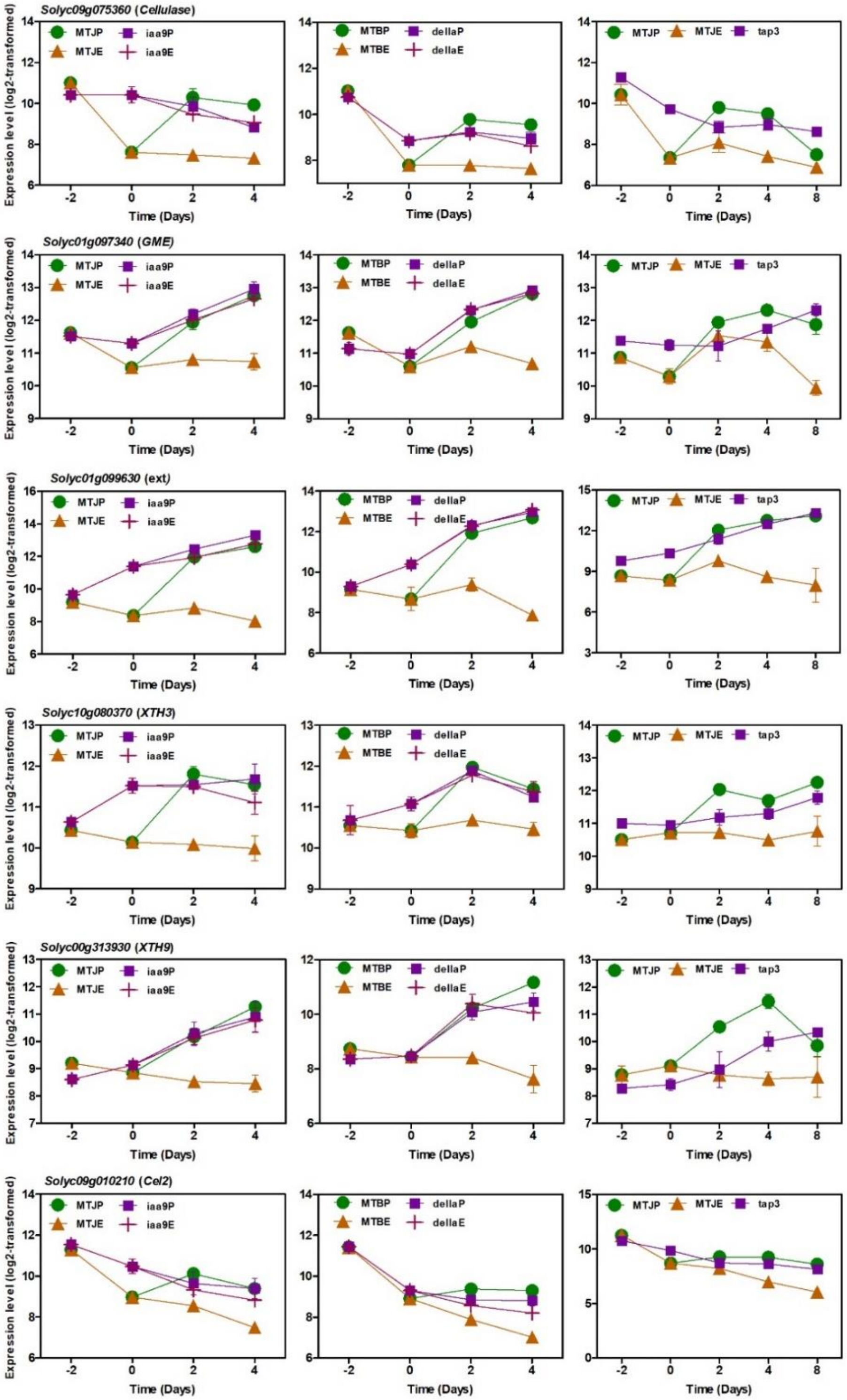

Figure S4 (continued).

Abbreviations: GME, GDP-mannose 3’,5’-epimerase; ext, endo-xyloglucan transferase; XTH3, xyloglucan endotransglucosylase-hydrolase3; XTH9, xyloglucan endotransglucosylase-hydrolase9; and Cel2, endo-1,4-beta-glucanase precursor.

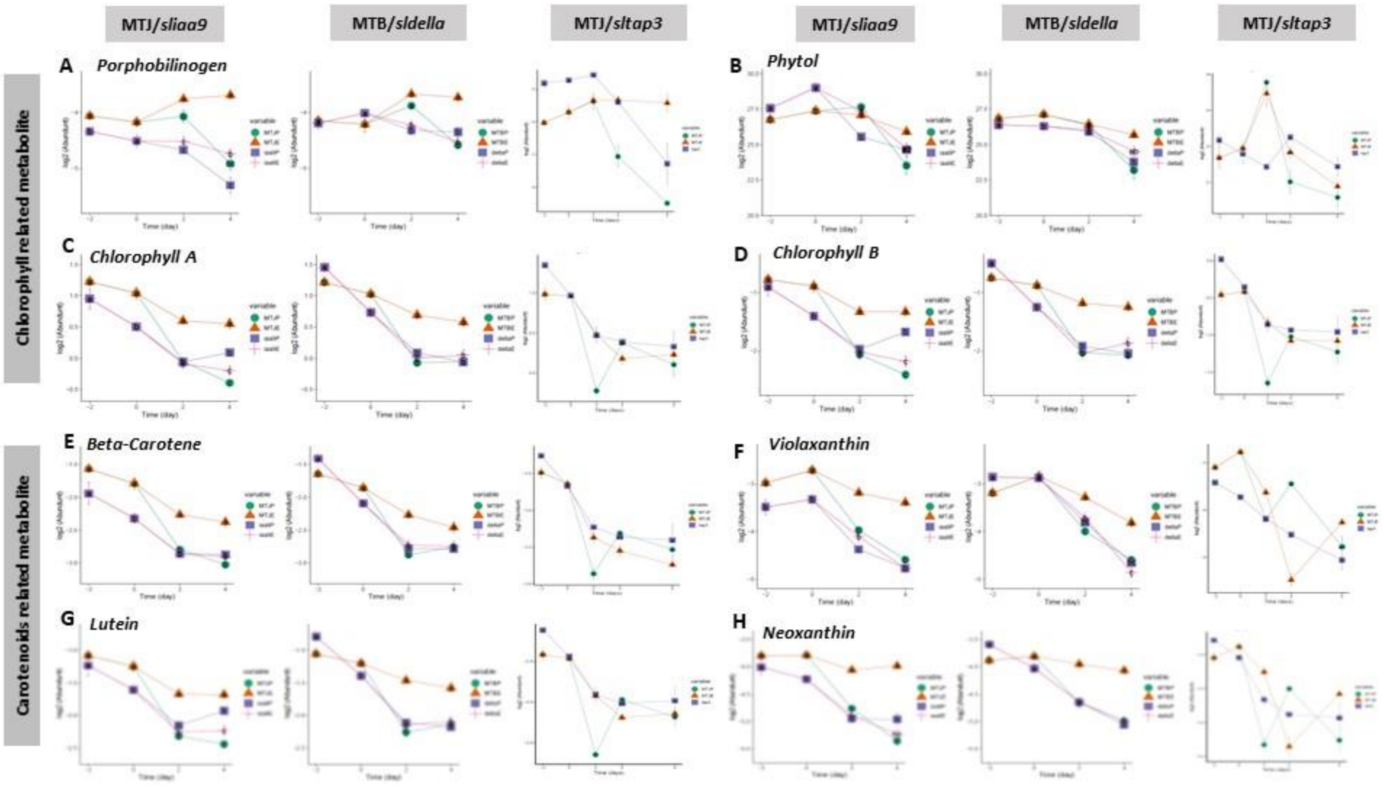

Figure S5. Changes of the photosynthesis- and carotenoid-related metabolites during fruit setting in MTJ/*sliaa9*, MTB/*sldella* and MTJ/*sltap3.*

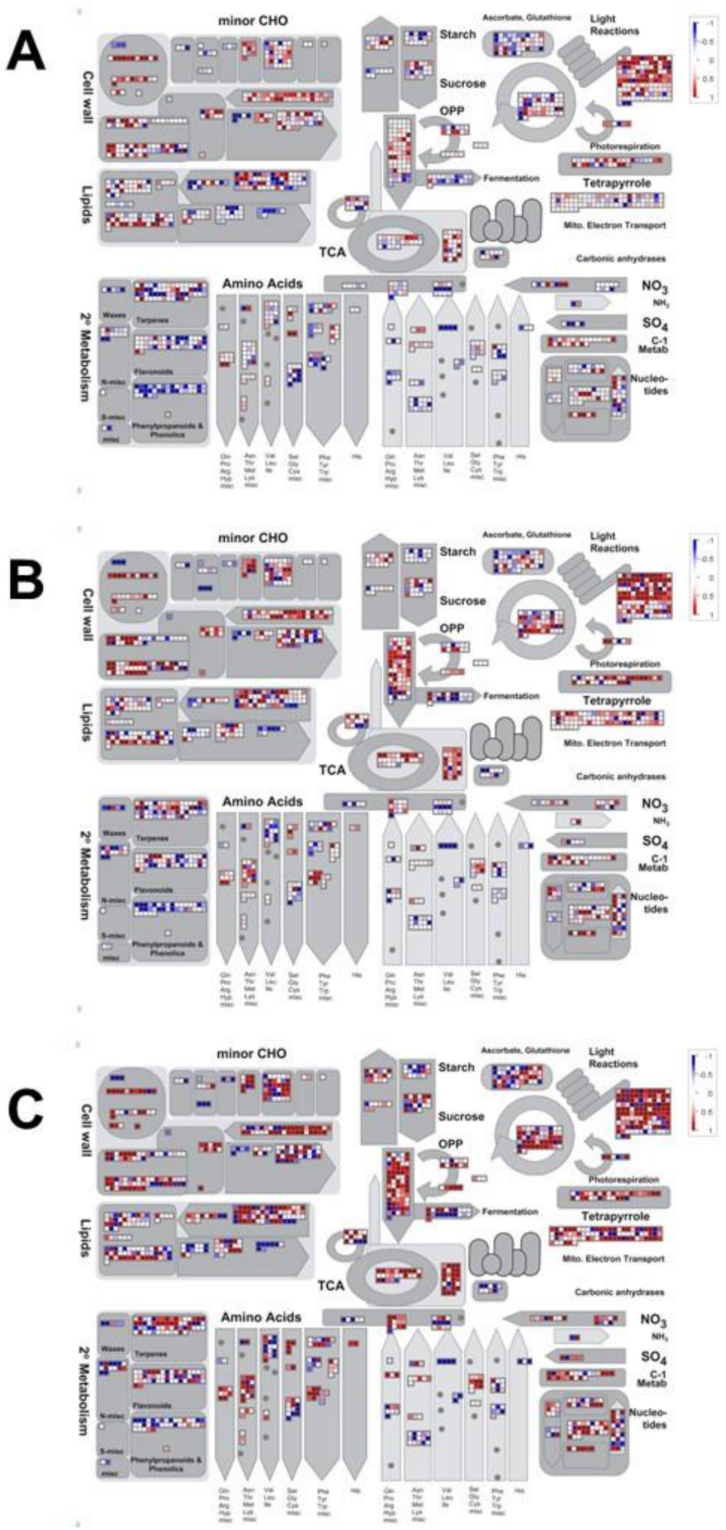

Figure S6. MapMan visualisation of DEGs in the profiles of MTJ/*sliaa9* at 0DAF (A), 2E-DAF (B) and 4E-DAF (C).

Significant, q-value ≤ 0.05; $|\log_2 FC| \geq 1$.

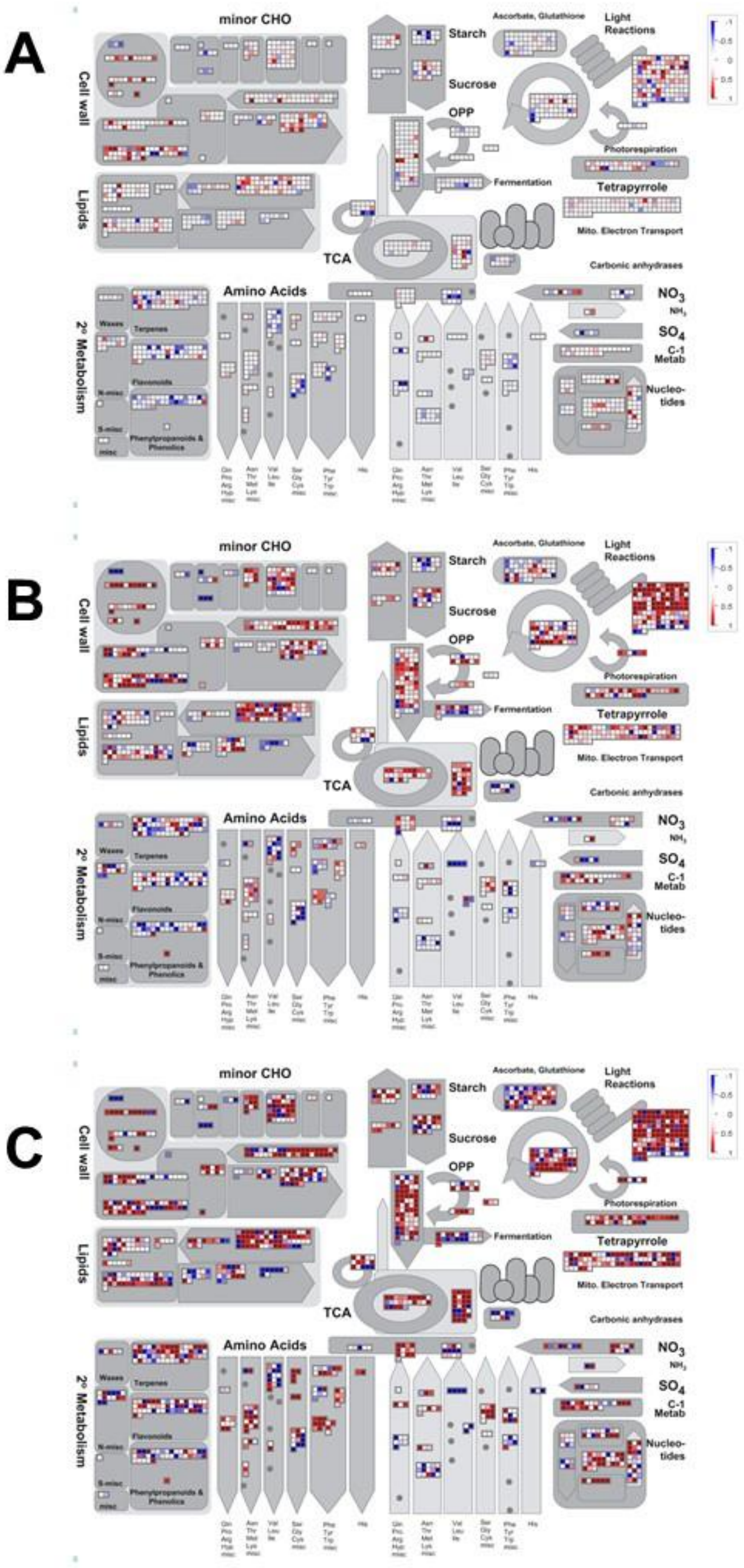

Figure S7. MapMan visualisation of DEGs in the profiles of MTJ/*sldella* at 0DAF (A), 2E-DAF (B) and 4E-DAF (C).

Significant, *q*-value ≤ 0.05; $|\log_2 FC| \geq 1$.

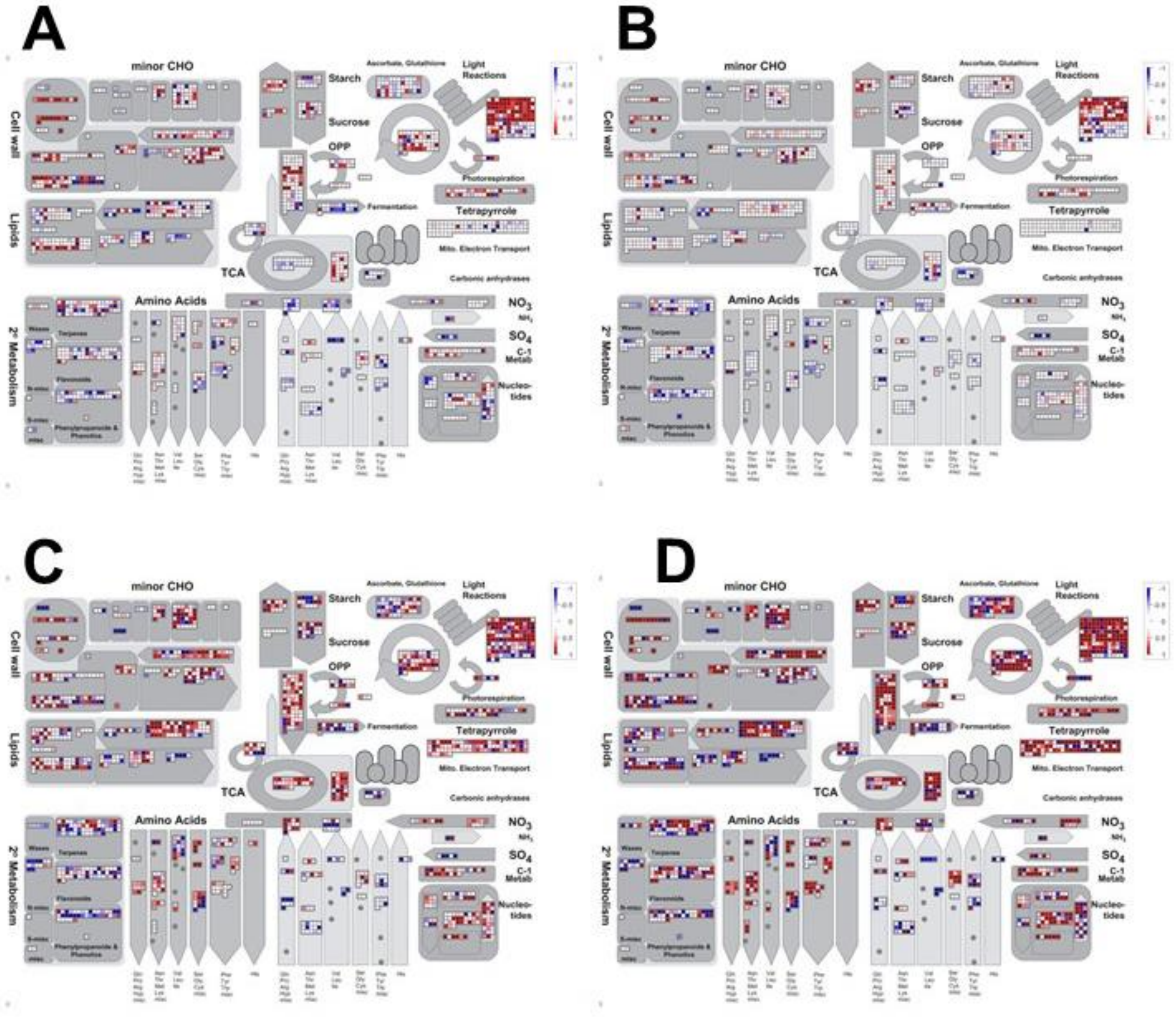

Figure S8. MapMan visualisation of DEGs in the profiles of MTJ/*sltap3* at 0DAF (A), 2E-DAF (B) and 4E-DAF (C).

Significant, *q*-value ≤ 0.05; $|\log_2 FC| \geq 1$.

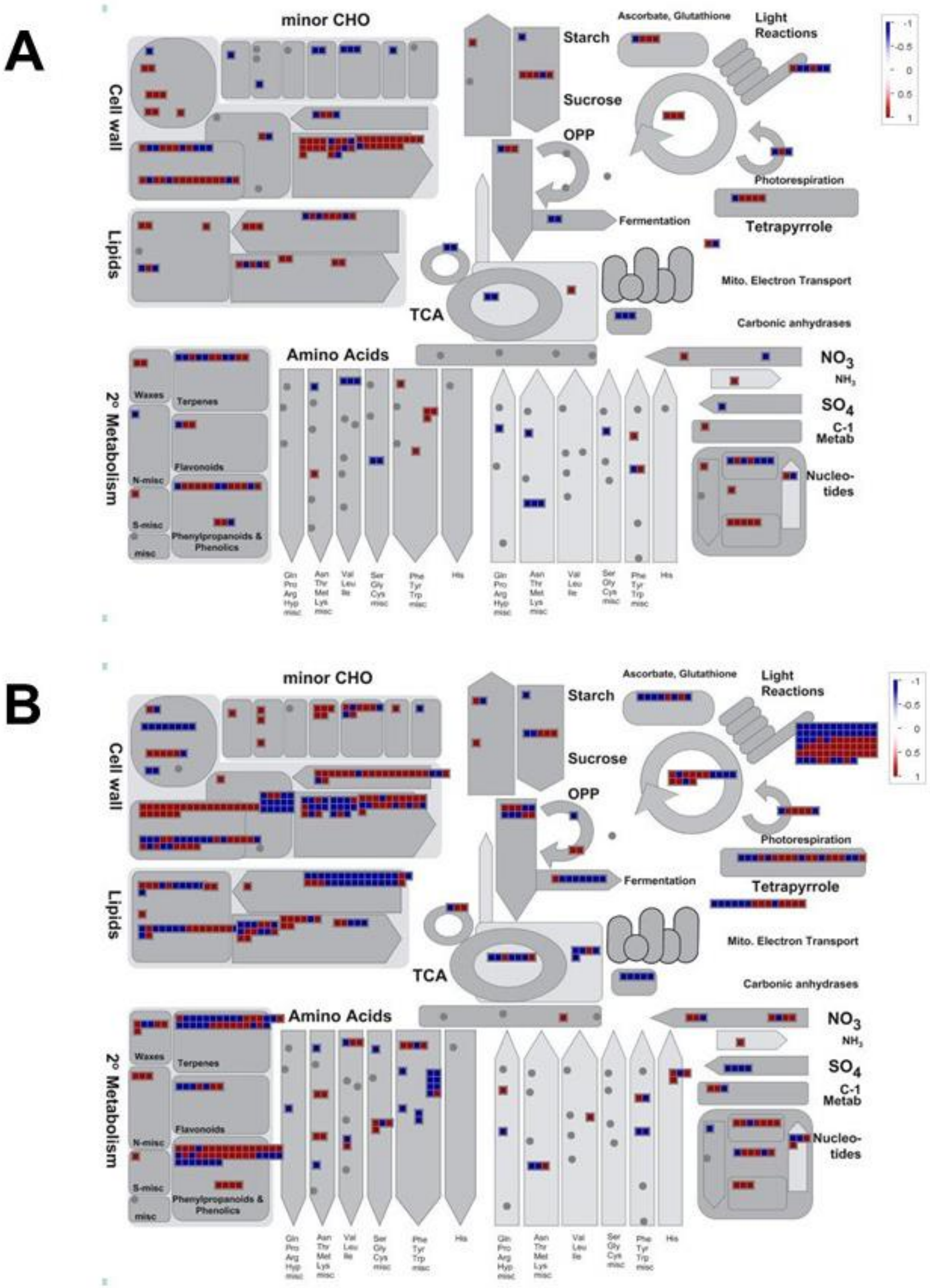

Figure S9. MapMan visualisation of DEGs in the profiles of Severianin/M82 at 0DAF (A) and 2E-DAF (B).

Significant, *q*-value ≤ 0.05; $|\log_2 FC| \geq 1$.
