# Supplementary material for: Transcriptomic, Hormonomic and Metabolomic Analyses Highlighted the Common Modules Related to Photosynthesis, Sugar Metabolism and Cell Division in Parthenocarpic Tomato Fruits during Early Fruit Set
Source: Cells. 2022 Apr 22;11(9):1420. doi: 10.3390/cells11091420 (PMC9102895; doi:10.3390/cells11091420)
Supplement: Supplementary file 1 [file cells-11-01420-s001.zip › Supplementary Methods S1.pdf]

Methods S1. Metadata of multi-omics analysis.

Materials and Methods

1.1 Growing conditions and measurement methods

1.1.1 Plant materials and growth conditions

In this study, Japanese (MTJ) and Brazilian (MTB) varieties of Micro-Tom were used as wild type of tomato (*Solanum lycopersicum*), and *sliaa9* with MTJ background, *sldella* with MTB background and *sltap3* with MTJ background were used as mutants generated by ethyl methanesulfonic acid (EMS) treatment. Seeds were absorbed overnight in deionized water and stored at 25°C for 4 to 6 days on deionized water-moistened filter paper under a photoperiod of 16 h/8 h with a light intensity of 100 $\mu molm^{-2}s^{-1}$. Tomato seedlings were transplanted into Rockwool cubes (75 × 75 × 65 mm) and grown in a nutrient solution with an electrical conductivity (EC) of 1.6 dSm^{-1} (Otsuka A, Otsuka Chemical Co., Ltd., Osaka, Japan) under fluorescent light with a photoperiod of 16 h/8 h and a light intensity of 300 $\mu molm^{-2}s^{-1}$ under light conditions of 25°C in bright conditions and 20°C in dark conditions. The experimental treatment was divided into two wards: one was manually pollinated on the day of flowering and another was manually emasculated. Day After Flowering (DAF) was used as a reference for the sampling of pistils from each line at two days before flowering (–2DAF, flowering day (0DAF), 2 days after flowering (2DAF), 4 days after flowering (4DAF) for the three mutants with corresponding WT and 8 days after flowering (8DAF) for the *sltap3* mutant and MTJ. Sampled ovaries were weighed and subsequently frozen at -80°C in liquid nitrogen until use.

The 'Severianin' and 'M82' cultivars were grown in nutrient culture in a coconut–peat substrate fed with hydroponic solution (revised-A nutrient prescription, Otsuka Chemicals, Osaka, Japan) with an EC of 0.8-1.5 ms cm^{-1}.

1.1.2 Transcriptome analysis using microarrays for the three mutants

For the quality check of extracted RNA, Agilent RNA6000 Nano LabChip® kit was used for 1 μl of 250 ng/3 μl RNA. The quality check was performed using an Agilent 2100 Bioanalyzer (Agilent) according to the kit protocol. Labeled cRNA was then purified for the same concentration of RNA using the Gene Chip® 3` IVT Express Kit (Affymetrix) according to the kit protocol. The concentration was measured with Nano Drop (Thermo Fisher) and sufficient concentration was obtained. Labeled cRNA was prepared to 15 μg/32 μl and fragmented according to the kit protocol. After that 80 μl of fragmented and labeled cRNA and hybridization cocktail mixture was injected into the GeneChip® Tomato Genome Array and incubated at 45°C at 60 rpm for 16 h according to the protocol. The chips of the microarray were set into the GeneChip® Fluidics Station 450 (Affymetrix) and the chips were washed and stained according to the accompanying manual. Finally, the Gene Chip scanner 3000 (Affymetrix) was used to scan the Chip and quantify the gene expression level.

1.1.3 RNA-seq analysis for Severianin and M82

Ovaries of Severianin and M82 (100 mg fresh weight (FW)) were collected in the green house located at Nasushiobara, Tochigi, Japan. We ordered Genome sequencing and RNA-seq analysis to BGI JAPAN (the Beijing Genomic Institute, Kobe, Japan). Illumina-based RNA-Seq analysis were performed to monitor genome-wide transcription changes in fruits of tomato parthenocarpic mutants and the corresponding control plants by ordering BGI Japan. Six illumia paired-end reads were generated for 'severianin.' In our pre-processing part, trim_galore tool (https://www.bioinformatics.babraham.ac.uk/projects/trim_galore/) was used for the adapter trimming and read pre-process with default parameters. High-quality filtered reads were mapped to tomato reference genome (the release of version SL4.0 of the tomato genome and version ITAG4.0 of the annotation) (https://solgenomics.net/organism/Solanum_lycopersicum/genome/) using Hisat2 with default parameters [1]. Using SAM tools, the resultant read mapped files (SAM format) were converted into sorted BAM files [2]. After that, a BAM index file was generated. These BAM files were processed using feature Counts to create raw count data [3].

1.1.4 Sample preparation for metabolome and hormonome analyses

Processing and extraction of freeze-dried samples in 2 ml tube was homogenized with a 5 mm zirconia bead in a Mixer Mill (Retsch, Haan, Germany) for 1 min at 20 Hz. After homogenized, samples were weighed for GC-MS, LC-MS, lipids, hormonome and LC-PDA analyses, respectively.

1.1.5 Non-targeted analysis of metabolites

1.1.5.1 GC-MS analysis

Metabolites were extracted from each sample at a concentration of 2.5 mg dry weight (DW) tissue per ml of extraction solution (methanol/chloroform/water = 3:1:1, v/v/v). Extraction and derivatization were performed as described [4,5]. Briefly, extracted samples were evaporated and dried with SPD2010 SpeedVac® from ThermoSavant (Thermo electron corporation, Waltham, MA, USA). For Methoxylation, 30 µl of methoxyamine hydrochloride (20 mg/ml in pyridine) was added to the sample and derivatized at room temperature for 24 h. Then, the sample was trimethylsilylated for 1 h using 30 µl of *N*-methyl-*N*-trimethylsilyltrifluoroacetamide (MSTFA) at 37°C with shaking the sample. After silylation was completed, 30 µl of *n*-heptane was added. The above derivatization was performed in a vacuum glovebox VSC-1000 (Sanplatec, Japan) filled with 99.9995% (G3 grade) dry nitrogen. Metabolites were detected using a GC instrument (Model 6890, Agilent Technologies, Palo Alto, CA USA) fitted with a Rxi-5Sil MS column (30 m × 0.25 mm inner diameter fused silica capillary column with a 0.25-µl film; Restek, Bellefonte, USA) coupled to a TOF mass spectrometer (LECO, St. Joseph, MI, USA). Helium (G1 grade) was used as a carrier gas at a constant flow rate of 1 ml/min and the temperature was maintained at 80ºC for 2 min and

then increased from 30ºC to 320ºC for 3.5 min. The transfer line and ion source temperatures were set at 250°C and 200°C. Data were acquired by a Pegasus IV time-of-flight mass spectrometer (LECO, St. Joseph, MI, USA) at a frequency of 30 spectra per second in the range of mass-to-charge ratio *m*/*z* = 60-800. Alkane standard mixtures (C8-C20 and C21-C40) purchased from Sigma Aldrich (St. Louis, USA) were used to calculate the retention index (RI). The resolved MS spectra were matched against reference mass spectra by using the National Institute of Standards and Technology (NIST) mass spectral search program for the NIST/EPA/NIH mass spectral library (version 2.0) and our custom mass spectral search software written in JAVA. The extracted MS spectra were finally identified or annotated according to their RI and comparison with the reference mass spectra in the libraries.

For quality check, methyl stearate was injected into each of the six samples. The data were normalized using the CCMN algorithm [6]. All raw data in NetCDF format were pre-processed by MATLAB (Mathworks, Natick, MA, USA). Data processing was described by [7].

1.1.5.2 LC-MS analysis

Extraction conditions for LC-MS: After preparation to 3 mg DW per 150 µl of extraction solution (methanol/water = 4:1, 2.5 M lidocaine and 2.5 M 10-camphour sulfonic acid), each sample was extracted using Retsch Mixer Mill MM310 at a frequency of 18 Hz for 7 min at 4°C. After centrifugation at 17,800 × g for 10 min, the supernatant was filtered using an Oasis® HLB µElusion plate (Waters Co., Massachusetts, USA) that had been conditioned with 200 l of methanol and equilibrated with 200 l of methanol/water (4:1, v/v) 0.1% acetic acid.

LC-q-TOF-MS assay conditions: Samples (1 µl) were analyzed using LC-MS (HPLC, Waters Acquity UPLC system; MS, Xevo G2 Waters Q-Tof) equipped with an electrospray ionization (ESI) device.

The analysis conditions: UPLC column: Acquity bridged ethyl hybrid (BEH) C18 (pore size 1.7 µ m, length 2.1 × 100 mm, Waters). Solvent system: Solvent A (water (HLPC grade) containing 0.1% formic acid) and Solvent B (acetonitrile containing 0.1% formic acid). Gradient program: 99.5% of A/0.5% of B at 0 min, 99.5% of A:0.5% of B at 0.1 min, 20% of A:80% of B at 10 min, 0.5% of A/99.5% of B at 10.1 min, 0.5% of A/99.5% of B at 12.0 min, 99.5% of A/0.5% of B at 12.1 min and 99.5% of A/0.5% of B at 15.0 min. Flow rate: 0.3 ml/min at 0 min, 0.3 ml/min at 10 min, 0.4 ml/min at 10.1 min, 0.4 ml/min at 14.4 min and 0.3 ml/min at 14.5 min. Temperature: 40°C. For detection in MS, the capillary voltage is + 3.0 kV, in positive mode and -2.75 kV in negative mode, the cone voltage is 25 V, the source temperature is 120°C and the desolvation temperature is 450°C. Cone gas flow rate was 50 l/h and desolvation gas flow rate was 800 l/h. Collision energy was 6 V. Detection mode was specified as *m*/*z* = 50-1500, scan duration time 0.1 s, inter-scan delay 0.014 s, and centroid. Data were recorded using MassLynx version 4.1 (Waters).

Data processing: Data matrices were generated using MarkerLynx (Waters) and Progenesis CoMet (Nonlinear Dynamics). The original peak intensity values were divided by the internal standard values to normalize the peak intensity values between the metabolite profile data.

1.1.5.3 Lipids, carotenoids and chlorophyll analysis

Sample extraction: Seven mg DW of sample was used for the analysis. Samples were allowed to stand at room temperature for at least 30 min and then 165 times the sample weight of the extract (chloroform/methanol = 1/2 (IS: beta-apo-8'-carotenal 12.5 uM and 1,2-dioctanoyl-*sn*-glycero-3-phosphocholine 1.25 uM and dibutylhydroxytoluene 0.1%) was added. The mixture was shaken for 5 minutes and then mixed again, followed by the addition of 44 times the volume of water to the sample weight. After mixing, it was centrifuged at 10000 × g in a high-speed centrifuge for 10 min at room temperature, 263 μl of chloroform and 263 μl of water were added to 1000 μl of the supernatant, and the mixture was left on ice for more than 10 min. Then, 340 μl of the organic phase was collected after centrifuging at 1000 × g for 3 min at 4°C in a high-speed centrifuge. This was concentrated and dried with SPD2010 SpeedVac® (Thermo Fisher Scientific, USA), mixed with 81 μl of chloroform/EtOH (1:1, v/v) containing 0.1% dibutylhydroxytoluene, and sonicated for 2 min. Then, it was centrifuged in a high-speed centrifuge at 10000 × g for 15 min at 4°C, and the supernatant was used for analysis.

LC-MS conditions for lipid analysis: One μl of extracts were analyzed using an LC-MS system (HPLC, Waters Acquity UPLC system; MS, Waters Xevo G2 Qtof) with an electrospray ionization (ESI) interface. Two solvents (A and B) were used to separate the solvents. The composition of the solvents was as follows: solvent A; acetonitrile: water: 1 M ammonium acetate: formic acid = 158 g: 800 g: 10 ml: 1 ml; solvent B; acetonitrile: 2-propanol: water: 1 M ammonium acetate: formic acid = 79 g: 711 g: 10 ml: 1 ml. The composition of the solvents was as follows: solvent A; acetonitrile: water: 1 M ammonium acetate: formic acid = 158 g: 800 g: 10 ml: 1 ml; solvent B; acetonitrile: 2-propanol: water: 1 M ammonium acetate: formic acid = 79 g: 711 g: 10 ml The analytical conditions were HPLC: column, Acquity UPLC HSS T3 (pore size 1.8 μm, 1.0 i.d × 50 mm, Waters), and the concentration of A:B was 35% B at 0 min, 70% B at 3 min, 85% B at 7 min, 90% B at 10 min, 90% B at 12 min, and 35% B at 12.5 min. The concentration of A:B was set at 35% B at 0 min, 70% B at 3 min, 85% B at 7 min, 90% B at 10 min, 90% B at 12 min, and 35% B at 12.5 min. Flow rate: 0.15 ml/min. Temperature: 55°C. For detection in MS, the capillary voltage is + 3.0 keV, cone voltage is 20V in positive mode and 40V in negative mode, source temperature is 120°C and desolvation temperature is 450°C. Cone gas flow rate was 50 l/h, desolvent gas flow rate was 450 l/h, collision energy was 6 V. The detection mode was specified as m/z = 100-2000, detection time 0.5 s, and centroid. Scans were repeated with one run as 15 min, and data were recorded using MassLynx version 4.1 (Waters).

LC-PDA conditions for Quantification of carotenoids and chlorophylls: Extracts (2 μl) were analyzed using an LC-photodiode array (PDA) (LC, Shimadzu LC-20AD system; PDA, Shimadzu SPD-M20A) operated by Shimadzu LCMS solution software (version 5.41). A two-solvent system was used to separate each carotenoid and chlorophyll. The analytical conditions were as follows: Column, Develosil C30-UG (2.0 mm I.D., 250 mm long; Nomura chemical); solvent A, methanol (4.5% water and 0.1% triethylamine); solvent B, hexane/isopropyl alcohol (60:40, v/v. 0.1% triethylamine); gradient program, 1% B at 0 min,

1% B at 9 min, 6% B at 9.5 min, 7% B at 19 min, 18% B at 20 min, 20% B at 18 min, 34% B at 28 min, 48% B at 36 min, 100% B at 48.5 min, 100% B at 53.5 min, 1% B at 54 min (total run time, 60 min); flow rate, 0.37 ml/min; column temperature, 30°C.

1.1.6 Quantitative analysis of plant hormones and their derivatives

1.1.6.1 Hormonome analysis

Ten mg DW of sample was used for the analysis. Extraction and quantification of plant hormones were performed according to the method described previously [8,9]. Endogenous cytokinins were measured using ultrafast liquid chromatography (UPLC) and ODS columns (AQUITY UPLC BEH C18, 1.7 mm, 2.1 9 100 mm; Waters) combined with a tandem quadrupole mass spectrometer (qMS/MS) equipped with an electrospray interface (ESI; UPLC-ESI-qMS/MS, UPLC-Xevo TQ-S; Waters). From the procedure described in the previous study, the two solvent gradients, solvent A (0.06% acetic acid) and solvent B (methanol solution of 0.06% acetic acid), were separated at 0 min: 95% A 5% B, 5 min: 65% A 35% B, 8 min: 20% A 80% B, 10 min: 1% A 99% B, and 13 min: 95% A 5% B at a flow rate of 0.25 mL/min. The data were processed using MASSLYNX™ with QUANLYNX™ 4.1 (Waters).

Endogenous GA, SA, ABA and auxin were measured using UHPLC-Q-Exactive (ThermoFisher Scientific, USA) MS probe modification. GA, SA and ABA were separated from solvent A (0.05% formic acid) and solvent B (0.05% formic acid in acetonitrile solution) on an ODS column (AQUITY UPLC BEH C18, 1.7 m, 2.1 mm ×100 mm; Waters) at a flow rate of 0.25 ml/min, with the following concentration gradients. 0 min: 90% A 10% B, 20 min: 45% A 55% B, 21 min: 99% A 1% B, 21.1 min: 90% A 10% B, 25 min: 90% A 10% B. Auxin was separated from solvent A (0.06% acetic acid) and solvent B (0.06% acetic acid in methanol solution) on an ODS column (AQUITY UPLC HSS T3, 1.8 m, 2.1 mm × 100 mm; Waters) at a flow rate of 0.25 mL/min, followed by the following concentration gradients of solvent A (0.06% acetic acid) and solvent B (0.06% acetic acid in methanol solution). 0 min: 90% A 10% B, 8 min: 10% A 90% B, 9 min: 1% A 99% B, 9.1 min: 90% A 10% B, 12 min: 90% A 10% B. Data were processed using XCALIBUR™ 2.2 (Thermo Fisher Scientific, USA).

1.2 Data analysis methods

The following analyses were performed mainly using R (ver. 3.6.3), a statistical analysis environment.

1.2.1 Microarray data analysis

We summarized all CEL files of the Affymetrix GeneChip Tomato Genome Array by the robust multichip average (Irizarry et al., 2003) with the 'affy' package (Gautier et al., 2004). We then filtered out probe sets at risk of cross hybridization (*x_at and *s_at) and probe sets

with the prefix 'RPTR' or 'AFFX.' For the microarray dataset for *sliaa9* and MTJ plants, we used ComBat() function in the sva package [10] to remove the batch effects, because this dataset consists of samples from different batches (date).

1.2.2 Dimensionality reduction and visualisation through PCA

Dimension reduction and visualisation of the dataset by the principal component analysis were executed for gene expression and metabolite accumulation data. After verifying that there was no significant difference in the distribution between the samples, PCA was performed by using the pca() function of the pcaMethods [11] and ggplot2 packages was used to draw the figures; the sample labels were located at the centre of gravity of the three samples. Because experiments were conducted in 2013 for the *sliaa9* and *sldella* mutants and 2014 for the *sltap3* mutant. As a result, we did not perform PCA using combined data from the three mutants and its backgrounds to account for the risk of incorrect PCA results being derived from batch errors between experiments.

1.2.3 Functional estimation of genes by GO enrichment analysis and KEGG PATHWAY enrichment analysis

Two enrichment analyses were executed to assess the biological functions that each cluster contributes. The GO enrichment analysis valuated whether GO term, which describes a process/position/function common to organisms called Gene Ontology, was significantly present in a cluster. Similarly, the KEGG PATHWAY enrichment analysis searches for the location of the genes in the cluster in each partial pathway of the KEGG PATHWAY and then performs a significance test on the number of times the partial pathways are attached. We used the gprofiler2 package (ver. 0.1.7) [12] for GO enrichment analysis. For KEGG PATHWAY enrichment analysis, clusterProfiler package (ver. 3.12.0) was used. These analyses were performed to the clusters generated by the time series clustering described above. The version of the server of gProfiler [13] was 'e98_eg45_p14_ce5b097'. In clusterProfiler [14], the enrich KEGG function was used, where 'sly' tomato is specified for organism and "kegg" for keyType.

1.2.4 Time-series plots of transcripts and metabolites

Since it was necessary to generate many of line plots of gene expression and metabolites from time series data, we used ggplot2 to automate the graphing process. The results of the multiple comparisons using the Tukey-Kramer method were also written at the dots of each time point using we used a modified version of
https://github.com/vicruiser/tukey_test_plot/blob/master/tukey_function.R.

1.2.5
RNA-sequencing (RNA-Seq) analysis and identification of differentially expressed genes (DEGs)

We performed illumina-based RNA-Seq analysis to monitor genome-wide transcription changes in fruits of tomato parthenocarpic mutants and the corresponding control plants. Fifteen Illumina paired-end reads (DDBJ DRA Submission ID: DRA012573) were generated for variety "Severianin" (source of pat-2, parthenocarpic). In our pre-processing part, trim_galore tool (https://www.bioinformatics.babraham.ac.uk/projects/trim_galore/) was used for the adapter trimming and read pre-process with default parameters. High-quality filtered reads were mapped to tomato reference genome (the release of version SL4.0 of the tomato genome and version ITAG4.0 of the annotation) (https://solgenomics.net/organism/Solanum_lycopersicum/genome/) using Hisat2 (Kim et al. Nat Methods, 2015) with default parameters. Using SAMtools (Li et al. 2009), the resultant read mapped files (SAM format) were converted into sorted BAM files. After that, a BAM index file was generated. These BAM files were processed using featureCounts (Liao et al. 2014) to create raw count data. DEGs were identified from raw count data using TCC package (Sun et al. 2013). Significant changes was set > 2-fold change and FDR < 0.05.

1.2.6 Weighted correlation network analysis of transcriptome and metabolome datasets

To understand the responsive network structures in each mutant, we constructed an unsigned correlation linkage from transcriptomics and metabolomics data, mainly according to earlier reports[15,16]. First, we calculated the mean values from n replicates (n = 3 for microarray, hormonome and RNA-seq data and n = 5—6 for metabolomome data) that are the mean metabolite concentrations or the mean normalised transcript expression. Here the data matrix size is X features (rows) and 12 time points (columns). The correlation networks were constructed by the weighted gene correlation network analysis (WGCNA) package [17]. After ranking the features (i.e. genes and metabolites) by the median absolute deviation (MAD), we chose the top largest 600 features for WGCNA. The soft-thresholding power was used according to a scale-free topology with a fit index of 0.8. Based on the topological overlap matrix, structural network properties like modularity were computed based on the topological overlap matrix. The networks were visualised using the igraph package (https://igraph.org/).

REFERENCES

1. Kim, D.; Langmead, B.; Salzberg, S.L. HISAT: a fast spliced aligner with low memory requirements. *Nat Methods* **2015**, *12*, 357-360, doi:10.1038/nmeth.3317.
2. Li, H.; Handsaker, B.; Wysoker, A.; Fennell, T.; Ruan, J.; Homer, N.; Marth, G.; Abecasis, G.; Durbin, R. The Sequence Alignment/Map format and SAMtools. *Bioinformatics* **2009**, *25*, 2078-2079, doi:10.1093/bioinformatics/btp352.

3. Liao, Y.; Smyth, G.K.; Shi, W. featureCounts: an efficient general purpose program for assigning sequence reads to genomic features. *Bioinformatics* **2014**, *30*, 923-930, doi:10.1093/bioinformatics/btt656.

4. Kusano, M.; Fukushima, A.; Kobayashi, M.; Hayashi, N.; Jonsson, P.; Moritz, T.; Ebana, K.; Saito, K. Application of a metabolomic method combining one-dimensional and two-dimensional gas chromatography-time-of-flight/mass spectrometry to metabolic phenotyping of natural variants in rice. *J Chromatogr B Analyt Technol Biomed Life Sci* **2007**, *855*, 71-79, doi:10.1016/j.jchromb.2007.05.002.

5. Kusano, M.; Redestig, H.; Hirai, T.; Oikawa, A.; Matsuda, F.; Fukushima, A.; Arita, M.; Watanabe, S.; Yano, M.; Hiwasa-Tanase, K.; et al. Covering Chemical Diversity of Genetically-Modified Tomatoes Using Metabolomics for Objective Substantial Equivalence Assessment. *PLOS ONE* **2011**, *6*, e16989, doi:10.1371/journal.pone.0016989.

6. Redestig, H.; Fukushima, A.; Stenlund, H.; Moritz, T.; Arita, M.; Saito, K.; Kusano, M. Compensation for systematic cross-contribution improves normalization of mass spectrometry based metabolomics data. *Anal Chem* **2009**, *81*, 7974-7980, doi:10.1021/ac901143w.

7. Kusano, M.; Fukushima, A.; Tabuchi-Kobayashi, M.; Funayama, K.; Kojima, S.; Maruyama, K.; Yamamoto, Y.Y.; Nishizawa, T.; Kobayashi, M.; Wakazaki, M.; et al. Cytosolic GLUTAMINE SYNTHETASE1;1 Modulates Metabolism and Chloroplast Development in Roots1 [OPEN]. *Plant Physiology* **2020**, *182*, 1894-1909, doi:10.1104/pp.19.01118.

8. Kojima, M.; Sakakibara, H. Highly sensitive high-throughput profiling of six phytohormones using MS-probe modification and liquid chromatography-tandem mass spectrometry. *Methods Mol Biol* **2012**, *918*, 151-164, doi:10.1007/978-1-61779-995-2_11.

9. Kojima, M.; Kamada-Nobusada, T.; Komatsu, H.; Takei, K.; Kuroha, T.; Mizutani, M.; Ashikari, M.; Ueguchi-Tanaka, M.; Matsuoka, M.; Suzuki, K.; et al. Highly sensitive and high-throughput analysis of plant hormones using MS-probe modification and liquid chromatography-tandem mass spectrometry: an application for hormone profiling in Oryza sativa. *Plant Cell Physiol* **2009**, *50*, 1201-1214, doi:10.1093/pcp/pcp057.

10. Leek, J.T.; Johnson, W.E.; Parker, H.S.; Jaffe, A.E.; Storey, J.D. The sva package for removing batch effects and other unwanted variation in high-throughput experiments. *Bioinformatics (Oxford, England)* **2012**, *28*, 882-883, doi:10.1093/bioinformatics/bts034.

11. Stacklies, W.; Redestig, H.; Scholz, M.; Walther, D.; Selbig, J. pcaMethods—a bioconductor package providing PCA methods for incomplete data. *Bioinformatics* **2007**, *23*, 1164-1167, doi:10.1093/bioinformatics/btm069.

12. Kolberg, L.; Raudvere, U.; Kuzmin, I.; Vilo, J.; Peterson, H. gprofiler2 -- an R package for gene list functional enrichment analysis and namespace conversion toolset g:Profiler. *F1000Res* **2020**, *9*, doi:10.12688/f1000research.24956.2.

13. Raudvere, U.; Kolberg, L.; Kuzmin, I.; Arak, T.; Adler, P.; Peterson, H.; Vilo, J. g:Profiler: a web server for functional enrichment analysis and conversions of gene lists (2019 update). *Nucleic Acids Res* **2019**, *47*, W191-w198, doi:10.1093/nar/gkz369.

14. Wu, T.; Hu, E.; Xu, S.; Chen, M.; Guo, P.; Dai, Z.; Feng, T.; Zhou, L.; Tang, W.; Zhan, L.; et al. clusterProfiler 4.0: A universal enrichment tool for interpreting omics data. *The Innovation* **2021**, *2*, 100141, doi:https://doi.org/10.1016/j.xinn.2021.100141.

15. Ichihashi, Y.; Kusano, M.; Kobayashi, M.; Suetsugu, K.; Yoshida, S.; Wakatake, T.; Kumaishi, K.; Shibata, A.; Saito, K.; Shirasu, K. Transcriptomic and Metabolomic Reprogramming from Roots to Haustoria in the Parasitic Plant, Thesium chinense. *Plant and Cell Physiology* **2017**, *59*, 729-738, doi:10.1093/pcp/pcx200.

16. Ichihashi, Y.; Date, Y.; Shino, A.; Shimizu, T.; Shibata, A.; Kumaishi, K.; Funahashi, F.; Wakayama, K.; Yamazaki, K.; Umezawa, A.; et al. Multi-omics analysis on an agroecosystem reveals the significant role of organic nitrogen to increase agricultural crop yield. *Proceedings of the National Academy of Sciences* **2020**, *117*, 14552-14560, doi:10.1073/pnas.1917259117.

17. Langfelder, P.; Horvath, S. WGCNA: an R package for weighted correlation network analysis. *BMC Bioinformatics* **2008**, *9*, 559, doi:10.1186/1471-2105-9-559.
